# The Psychometric Structure of Executive Functions: A Satisfactory Measurement Model? An Examination Using Meta-Analysis and Network Modeling

**DOI:** 10.3390/bs13121003

**Published:** 2023-12-08

**Authors:** Kevin P. Rosales, Eugene H. Wong, Lisa Looney

**Affiliations:** Department of Child Development, California State University, San Bernardino, CA 92407, USA; ewong@csusb.edu (E.H.W.); lisa.looney@csusb.edu (L.L.)

**Keywords:** executive functions, latent variable modeling, network modeling, meta-analysis

## Abstract

A long-standing debate among cognitive scientists has focused on describing the underlying nature of executive functions, which has important implications for both theoretical and applied research. Miyake et al.’s three-factor model has often been considered the gold-standard representation of executive functions and has driven much research in the field. More recently, however, there have been increasing concerns that the three-factor model does not adequately describe a highly complex construct such as executive functions. The current project presents two studies that examine the veracity of Miyake et al.’s model and propose a new approach (i.e., network modeling) for detecting the underlying nature of executive functions. The current results raise questions about the psychometric strength and adequacy of the three-factor model. Further, the studies presented here provide evidence that network modeling provides a better understanding of executive functions as it better captures (relative to latent variable modeling) the complexity of cognitive processes. Theoretical and applied implications are discussed.

## 1. Introduction

A large body of research in cognitive psychology has devoted much attention to understanding the nature and structure of executive functions (EF). Generally, EF has been defined as elementary control processes (e.g., regulation of behavior, planning, self-monitoring, self-control, problem-solving, maintenance of attention) that guide complex behaviors often deemed important in various settings [1]. Executive functions have been shown to predict school success [2], school readiness [3], physical health [4], and intelligence [5].

The process of characterizing the structure of EF has been a long-standing focus of researchers in numerous fields [6,7,8]. Cognitive scientists have engaged in debates surrounding the generality (EF develops in a domain-general manner) versus the specificity of EF (EF develops according to context-specific demands) [6], but over the last couple of decades, the prevailing work has centered on EF as a set of component skills or processes that have predictive power for a variety of complex behaviors [9]. Specifically, Miyake and colleagues [9] identified a data-driven model that explained EF as composed of three components (i.e., updating, shifting, inhibition) that are both unified in their power to explain EF and diverse in their ability to operate individually. However, others have called this reductionist view of EF problematic, arguing that the components of EF cannot be conceptually or psychometrically condensed but instead must be considered within specific contexts [10]. What has ensued is an enhanced discussion about theoretical and conceptual issues related to EF, with calls for a renewed focus on theory development [7] and a balanced approach between competing models of EF (i.e., domain-general vs. context-dependent conceptualizations) [11].

This debate is arguably an important one, as understanding EF has important implications at both the theoretical and applied levels. Given the importance of EF for a variety of outcome variables [12,13], much work has been devoted to developing intervention strategies designed to mitigate EF deficits [14,15]. Mixed results in the findings produced by this intervention work [15,16] have also added to the debate about the nature and structure of EF, leading cognitive scientists to ponder whether mixed results are a function of interventions themselves or a result of not targeting the correct construct. While much of the focus of the debate has been centered on the conceptualization of EF, one could argue that the discussion about the conceptualization of EF has inevitably bled into the psychometric study of EF since the measurement of a construct is intricately tied to how one describes the concept. As such, one possibility to consider is whether the crux of the debate is less about the conceptualization of EF and more about the psychometric structure (operationalization) used to understand it.

As there is consensus in cognitive science that EF is significant for certain outcome variables [1], it is critical that the field has confidence that the accepted models of EF constructs are accurate for both theoretical and applied purposes. As researchers apply work on EF in real-world settings (e.g., school environments) to inform treatment and intervention decisions for individuals, ensuring an accurate representation of the constructs enhances the efficacy of applied methods. Though the research on EF is extensive, minimal work has focused on evaluating the strength and veracity of the psychometric model of EF. The current paper (utilizing two studies) is focused on enhancing our understanding of the psychometric structure of EF for these purposes. 

### 1.1. Three-Factor Model of Executive Function (Miyake et al. [9])

The most prominent model of EF used in the field of cognitive psychology is the three-factor model [9]. This latent variable model proposes a theoretical account for the structure of EF, using the manifest variables of shifting (i.e., the ability to switch between different mental states; cognitive flexibility), updating (i.e., holding information in working memory while subsequently replacing the information with relevant new information), and inhibition (i.e., the ability to suppress distracting information while engaging in an on-going task). These three variables were considered key for the model, given their predictive role in other higher-order cognitive processes [9]. Exploratory factor analysis (EFA) and confirmatory factor analysis (CFA) revealed that the data yielded and fit a specified three-factor structure of executive functions [9]. Further, the three factors (i.e., shifting, updating, and inhibition) were correlated, with coefficients ranging from small to moderate [9]. These findings (i.e., EFA and CFA yielding a distinct three-factor structure, yet the factors were correlated) led Miyake et al. [9] to argue for the general idea of both unity and diversity of EFs.

Since Miyake and colleagues [9] introduced their findings to the field, many cognitive scientists have adopted the model as the “gold standard” of EF (see [7] for a more complete overview), leading subsequent research to use this model to understand and measure EF [5,17,18], as well as to help explain the relationship between EF and other variables [19,20]. However, recent work has begun to explore alternative ideas as scientists ponder the best ways to understand the complexity of EF as a construct [7].

### 1.2. Is the Three-Factor Model of EF Acceptable?

The Miyake and colleagues [9] three-factor model uses a latent variable model approach, thereby relying on manifest (or directly measured or observable) variables to assess whether the latent (or unobserved) variable is present. Historically, cognitive ability research has predominantly used this latent variable modeling approach to explore the structure of EF. However, more recent work has called into question the effectiveness of this approach for numerous reasons.

First, both the original Miyake et al. [9] study and others that have used the model (e.g., [5,19]) have yielded findings in which more than half of the variance in the manifest variables is unexplained by the latent factor. While the model showed a good fit, the variance explained across all EF manifest variables was, at best, modest [5,9,19], indicating that the measurement model is not entirely satisfactory. These findings and those of others [18,21,22] call into question the construct validity of the EF latent construct. Thus, it is important to systematically examine (via meta-analysis) whether studies using the three-factor model of EF consistently show poor measurement models of EF and how this might theoretically argue for a reconsideration of the model moving forward.

Second, latent variable models present the disadvantage of relying on subjective interpretations of the latent factors, with the decision for how to define the latent factors residing with the researchers [23,24]. This is problematic given that measures of cognitive ability are often not process pure and thus, confining such measures to a latent factor that is labeled by the researcher can potentially misrepresent the true nature of the constructs. 

Finally, one of the most significant disadvantages of latent variable modeling lies in the principle of local independence, which states that observations explained by one latent factor are independent of observations explained by another. In other words, latent variable models enforce the idea that manifest variables cannot correlate with one another [25]. This poses an inherent problem in EF research as it assumes that a task in one EF component (e.g., working memory) cannot bleed into any other component (e.g., shifting), which is largely unrealistic given the complexity of the construct.

In short, while latent variable approaches have dominated much of our understanding of EF, the inherent problems with this method warrant discussion of its viability. The longstanding debate about the nature and structure of EF demonstrates the field’s understanding of the complexity of the construct itself. Arguably, this debate (and the psychometric approaches used to understand it) is related to the established division in the field of cognitive science: those that study the mechanisms of cognition and those that study the individual differences in cognition [26]. Utilizing psychometric approaches that can bridge this divide and allow cognitive science to address both approaches would be worthwhile. While latent variable modeling is well-developed statistically and is useful as a modeling technique [26], its approach does not allow cognitive scientists to dive deeper into the complexity of EF components—a must for understanding more context-dependent models of EF.

### 1.3. Network Analysis: An Alternative Approach

One alternative to increasing our understanding is utilizing network analysis of EF. Network models can surpass the limitations of latent variable models (e.g., the subjectivity of the latent factors, principle of local independence) by detecting the underlying structure of cognitive abilities in an exploratory manner [25]. Kan et al. [25] noted that network models do not suffer from any of the limitations held by latent variable models for several reasons. 

First, network modeling does not require researchers to specify latent factors, as there is no common cause for the manifest variables in a study. Therefore, the techniques used in network modeling eliminate the subjectivity found in CFA models of cognitive abilities. Second, network modeling is not constrained by the principle of local independence; therefore, tasks in a study are allowed to freely form connections with one another. Tasks that share similar processes will cluster more closely than tasks in the model that might not share many processes—an approach that is more compatible with modern views of cognitive abilities. Specifically, more contemporary ideas in the field recognize the complexity of the cognitive system and state that cognitive abilities frequently overlap during cognitive activities [27]. Therefore, network modeling is an approach consistent with the idea that cognitive tasks are not process pure. Taken together, these considerations provide strong arguments for the use of network modeling over latent variable modeling, especially in situations where the tasks of choice are theoretically driven (e.g., complex span tasks).

### 1.4. The Current Project

This project addresses a significant gap in the cognitive-psychological literature that has both theoretical and practical implications. A coherent model of EF that accounts for acceptable levels of variance in manifest variables of the construct informs basic research on cognitive processes as well as applied work in a variety of settings in which understanding cognitive abilities is important (e.g., the school setting). At the theoretical level, the availability of an EF model that better represents the observable cognitive processes will guide future work that seeks to explain the nature of EF. Alternatively, an enhanced model of EF has important implications in applied research that often focuses on intervention efficacy; that is, by having a model with greater explanatory power, professionals can better target specific skills for intervention and likely develop more effective strategies to remediate these abilities. Across two studies, this paper will first test the psychometric strength and adequacy of the currently accepted model of EF proposed by Miyake et al. [9] using meta-analysis. In the second study, a new model of EF utilizing network modeling will be described. 

## 2. Study 1

Using the currently accepted model of EF [9], the goal of Study 1 is to examine the psychometric strength of a three-factor structure via a meta-analysis of ten studies.

### 2.1. Method

#### 2.1.1. Participants

Across ten studies, the sample consisted of 2478 participants, with a mean participant age of 28 years. Participants consisted of healthy children and adults, and all were proficient in English. 

#### 2.1.2. Selection of Studies

To obtain the studies used in this meta-analysis, a literature search of the following databases was performed: Google Scholar, PsycInfo, and Academic Search Premier. The following key terms were used to search: (executive functions OR executive functioning) AND (latent variable analysis OR confirmatory factor analysis) AND (three-factor model of executive functions). The above-listed search terms were entered individually and in combinations that included: (executive functions) AND (latent variable analysis), (executive functions) AND (confirmatory factor analysis), (executive functioning) AND (latent variable analysis), (executive functioning) AND (confirmatory factor analysis), and (three-factor model of executive functions) AND (latent variable analysis), and (three-factor model of executive functions) AND (confirmatory factor analysis). Additionally, citations made in relevant papers found in this search were reviewed. Studies citing Miyake and colleagues’ [9] paper were reviewed. No backward searches were done on Miyake and colleagues’ [9] paper because the model of executive functions was first proposed in that publication. 

#### 2.1.3. Inclusion and Exclusion Criteria

The following criteria were used for study selection: (1) strict use of the three-factor model of executive functions (assessing shifting, updating, and inhibition) proposed by Miyake et al. [9]; (2) use of latent variable analysis or confirmatory factor analysis of the three-factor model of EF; (3) use of similar operational definitions of EFs to those used by Miyake et al. [9]; this allowed for the exclusion of studies that assessed executive function but did not operationalize EF in a manner consistent with cognitive psychology research. Thus, studies were selected if the following definitions were used: Inhibition defined as inhibition of dominant or prepotent responses; shifting defined as shifting between tasks or mental sets; and updating defined as updating or monitoring of working memory representations; (4) studies conducted with typically developing adults and children; that is, the primary studies included samples of children and adults without any diseases; (5) administration of at least two manifest variables (tasks) for each executive function construct; and (6) reporting of descriptive statistics, correlation tables, and tables of standardized path loadings for the model. 

#### 2.1.4. Procedure

**Figure 1:**
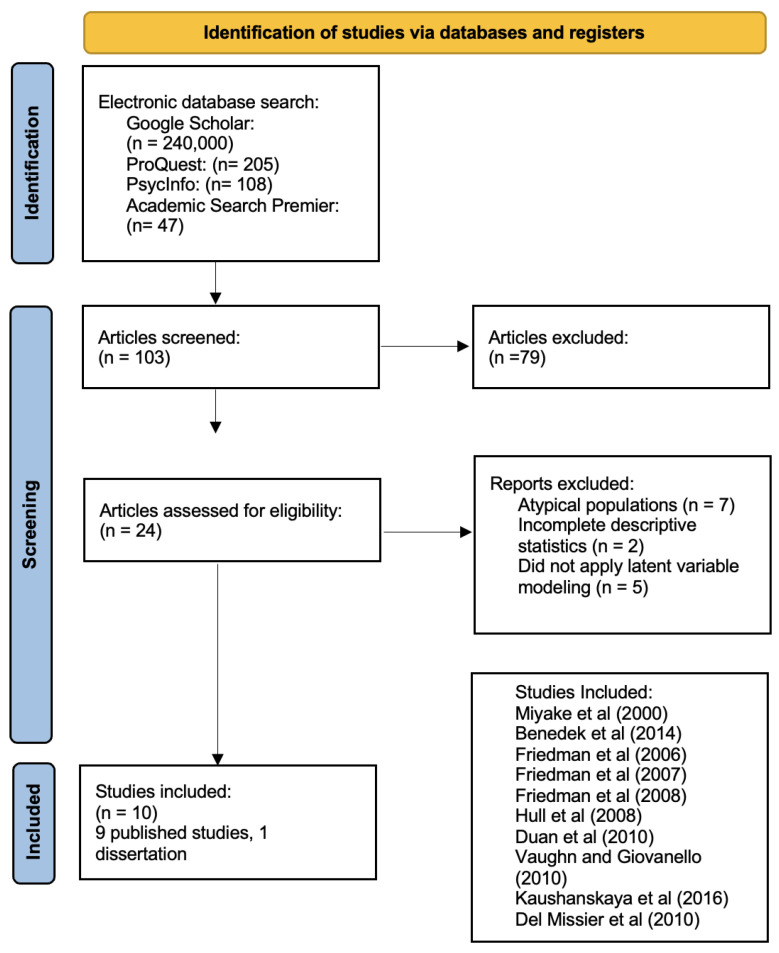
PRISMA-based flow chart illustrating the study selection process. Studies included: Miyake et al. [9], Benedek et al. [19], Friedman et al. [5], Friedman et al. [17], Friedman et al. [18], Hull et al. [21], Duan et al. [28], Vaughn and Giovanello [20], Kaushanskaya et al. [29], Del Missier et al. [30].

A broad search was performed using the databases and key terms outlined above. A preliminary sample of 24 studies was obtained. The titles and abstracts of studies were reviewed to determine their adequacy. After applying the criteria stated above, any studies that did not meet the criteria were removed from the sample. A set of 10 studies was deemed appropriate for Study 1; among these studies, nine were published studies and one was a dissertation. The rationale for the exclusion of studies included: (a) the study did not report complete descriptive statistics; (b) measures of executive functions were utilized but CFA analyses were not conducted; and (c) the study was conducted with an atypical population (e.g., patients with schizophrenia, children with ADHD, or patients with other neurological/neurodevelopmental disorders). See Figure 1 below.

#### 2.1.5. Data Analysis

Once the final sample of 10 studies was obtained, the studies were coded on different dimensions that included: type of study (publication vs. unpublished), year of publication, sample size, age group (children/adolescents vs. adults), mean age, and task type used to measure each latent variable. Before analysis, the standardized beta coefficients were transformed. Using SPSS software version 29, three meta-analytic analyses were performed (one per EF factor). In addition, the homogeneity of effect sizes and publication biases were assessed. In terms of homogeneity, both Cochran’s Q and I^2^ were calculated. For Cochran’s Q, a significant Q value indicated that there is a lack of homogeneity among the effect sizes. In terms of I^2^, a percentage of homogeneity is provided. The proportions range from 0 to 100%, with higher percentages dictating less homogeneity among effect sizes. A large amount of homogeneity would range from 0% to 25%; moderate homogeneity ranges from 25% to 50%; and small homogeneity ranges from 75% to 100%. Homogeneity represents the degree to which the effect sizes differ between studies. Higher I^2^ values indicate more inconsistency of effect size values across studies, while lower I^2^ values indicate greater consistency of effect size values across studies. Moderator analyses were conducted to determine if the effect sizes differed across (1) age groups: children vs. adults and (2) types of EF construct. 

### 2.2. Results

All standardized beta coefficients were transformed to Fisher’s Zr values using the Fisher’s r to Z transformation procedure across all 10 studies. This transformation was performed to normalize the distribution and stabilize the variance. The median loading of each factor was taken for each study (in the end, each study yielded three effect sizes that pertained to each factor). The median was chosen due to the high variability among standardized beta coefficients for each factor. It was thought that the median factor loading would be more resistant to this variability in comparison to the mean loading for each factor and provide a more accurate index of effect size. Ultimately, the mean effect size for the three factors of executive functions was tested across studies. Thus, three separate meta-analytic analyses were performed, one for each EF factor. In addition, all studies were weighted to correct for sample size differences among studies. 

**Figure 2:**
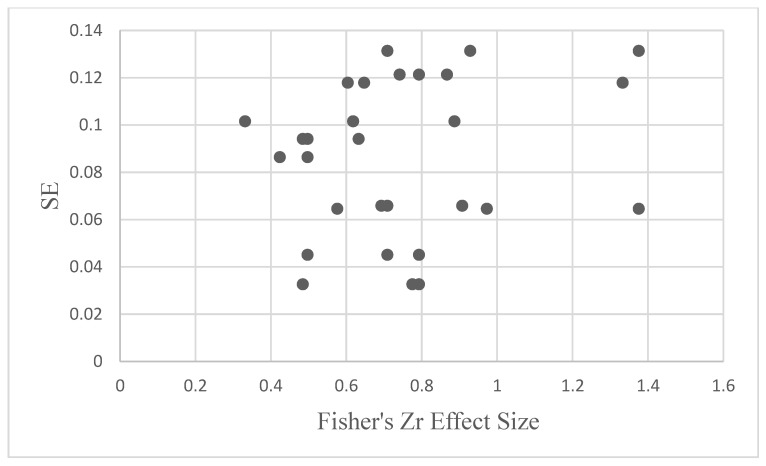
Funnel plot displaying standard errors as a function of effect size. N= 10 studies.

A random effects model was adopted, and the results were as follows for each EF model: For shifting, the mean effect size was 0.85, z = 16.76, *p* < 0.001. In shifting, the total amount of variance explained in the factor by the tasks was 0.72. For inhibition, the mean effect size was 0.55, z = 14.72, *p* < 0.001. The total amount of variance explained in inhibition by the inhibition tasks was 0.30. In updating, the mean effect size was 0.80, z = 9.66, *p* < 0.001. The amount of variance explained in the updating factor by the updating tasks was 0.64. See Table 1 for complete results and descriptive statistics. To determine the existence of publication bias, a funnel plot was created using the variation of effect size as a function of standard error. See Figure 2 for the funnel plot. 

### 2.3. Discussion

The goal of Study 1 was to evaluate the psychometric quality of the unity and diversity model of EF [9] via a meta-analysis. The results showed that across ten studies, the measurement models of EF were modestly acceptable, at best. For inhibition, shifting, and updating, the amount of unexplained variance across all studies was high, which indicates low explanatory power at the latent variable level. This is especially true for inhibition. The mean effect sizes were 0.85 for shifting, 0.55 for inhibition, and 0.80 for updating. The amount of variance explained ranged from 30% to 72%. There was a large inconsistency across the EF regarding explanatory power. In addition, the moderator analyses revealed that the size of the effect varied across the EF latent factors. However, the effect sizes did not significantly change between adults and children. 

The findings of Study 1 corroborate the results of earlier studies, which showed that there was a lack of adequate variance explained, as evidenced by the mean effect size indices when using the Miyake et al. model [5,19]. Thus, Study 1 highlights the inefficacy of this model’s use for understanding cognitive constructs. However, in contrast to the previous literature highlighted earlier, the results shown here in Study 1 have a greater weight of evidence due to the meta-analytic approach, which analyzes a sample of studies as opposed to individual studies. Given that Study 1 shows that the measurement model of EFs lacks adequate explanatory power, the goal of Study 2 was to examine the psychometric structure of EF using an alternative statistical approach (i.e., network analysis). The goal of this examination was to help determine the degree to which EFs are structured in a network fashion with other cognitive abilities. Network analysis as an alternative and more modern approach to statistically and theoretically analyzing cognitive abilities (in comparison to traditional latent variable modeling) could open the door to uncovering and describing the complexity of EF components that more traditional psychometric approaches [9] seem to not capture.

## 3. Study 2

In this second study, network analyses were conducted on the EF data utilized in Study 1. Doing so allowed for an alternative examination of the underlying psychometric structure of EF. This was done using the R software version 4.3.2 code from Kan et al. [25] and van der Mass et al. [26].

### 3.1. Method

#### 3.1.1. Participants

Participants in this study are the same as those described in Study 1. Given that this study utilized seven of the 10 studies from Study 1, the total sample size is N = 1836 here for Study 2. 

#### 3.1.2. Measures

EF measures in this study consisted of inhibition, shifting, and updating. See Table 2 for all measures included in network model analyses. For a more detailed description of the paradigms, please see [9,17,18,19,20,21]. 

#### 3.1.3. Statistical Procedure

##### Model Fit Evaluation for Psychometric Network Models

Study 2 followed model fit evaluation recommendations provided by previous literature [31]. Thus, model fit would be adequate when (a) the model chi-square to degrees of freedom ratio is less than or equal to 3.00, (b) the comparative fit index (CFI) and Tucker-Lewis Index (TLI) are greater than or equal to 0.95, and (c) Root Mean Square Error of Approximation (RMSEA) values are less than or equal to 0.06. 

##### Psychometric Analysis

Psychometric network models were conducted on correlation matrices from studies adopting the Miyake et al. [9] three-factor model of EFs. Analyses were conducted using the q graph and open MX packages in R. Results were visualized using a q graph. The network models were produced using the graphical least absolute shrinkage and selector operator (gLASSO) regularization technique. Two parameters were manually set: hyperparameter gamma (set to 0.50) and the tuning parameter lambda (set to 0.01) [26]. Setting the hyperparameter conservatively (as was done in this study) allows for the regularization technique to prefer simpler models with fewer edges. The lambda parameter settings set here allow for only the detection of true edges and not any spurious edges. 

### 3.2. Results

A subset (seven studies) of the studies used in Study 1 were analyzed here in Study 2. Only those studies that reported correlation matrices were included, as that is a requirement for conducting network analyses. Consequently, Kaushanskaya et al., Vaughn and Giovanello, and Friedman et al. were excluded from Study 2. Thus, a total sample of N = 1836 participants was included in Study 2. All included studies contained measures of updating, shifting, and inhibition. It is worth noting that network models are evaluated by the strength of the connections among the nodes. The nodes represent the cognitive tasks themselves, while the color of the nodes represents the construct they are intended to measure (i.e., updating, shifting, inhibition). The connections between the nodes are referred to as edges, which represent the partial correlations among the variables. The thickness of the edges indicates the strength of the partial correlations between any two given tasks (nodes), such that the thicker the edge, the stronger the partial correlation between two tasks, while the thinner the edge, the weaker the partial correlation.

As can be observed below, the general pattern of observation is that the network models of EFs show moderate to strong edges between tasks representing the same construct but little to no edge connections between tasks representing different EF constructs. These findings show that contrary to popular belief about the unity/diversity model of EF [9], there is adequate diversity between EF tasks but little to no unity among them. 

**Figure 3:**
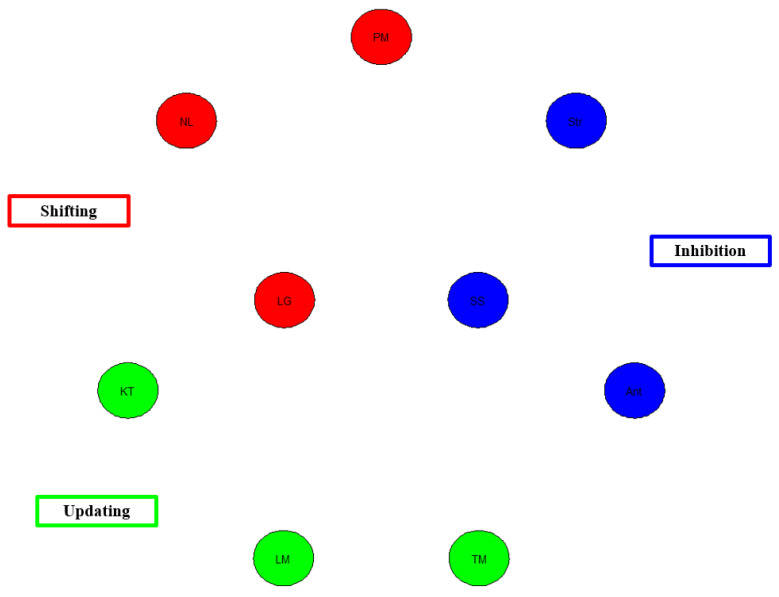
Network Model of Miyake et al. [9] EF data. Note. Red = shifting. NL = number-letter, PM = plus-minus, LG = local-global, Blue = inhibition. Str = Stroop test, Ant = anti-saccade, SS = stop-signal. Green = Updating. KT = keep-track, LM = letter memory, TM = tone monitoring.

Figure 3 shows the EF network model analysis using the reported correlation matrices from the Miyake et al. [9] study. As illustrated in the figure, the overall network model based on the Miyake et al. data shows no evidence of a network of EF abilities in this study. There is neither divergence nor convergence in the EF tasks. This is likely the case given the alarmingly low correlations between the tasks, as these ranged from 0.00 to 0.41 across all EF tasks.

In terms of model fit, there is poor model fit, χ^2^(36) = 122.47, *p* < 0.001, CFI = 0, TLI = 0, RMSEA = 0.13, AIC = 3508.07, BIC = 3534.35. CFI for the present model is below the acceptable threshold of 0.95, as is the case for TFI. In addition, the RMSEA value falls above the threshold of 0.06. Taken together, the Miyake et al. [9] network model shows poor model fit. 

**Figure 4:**
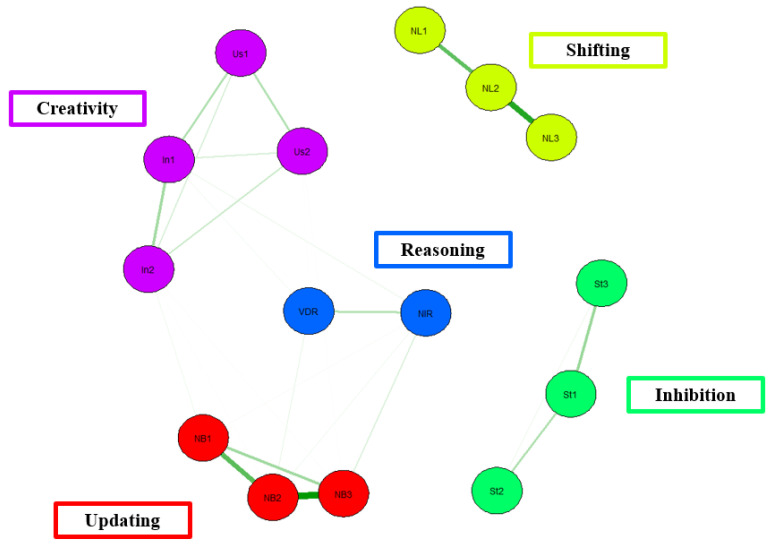
Network Model of Benedek [19] EF data. Note. Purple = Creativity. Us = unusual uses, In = instances task. Yellow = Shifting. NL = number-letter. Green = Inhibition. St = Stroop. Red = Updating. NB = n-back task. 1 = block 1, 2 = block 2, 3 = block 3.

Figure 4 shows a network model derived from the data reported in the Benedek [19] study. In this network model, the tasks used to measure EF are well connected within each EF domain. However, similar to the Miyake et al. [9] study, there is no convergence across EFs. That is, the inhibition tasks, updating tasks, and shifting tasks lack connections between one another. 

In terms of model fit, there is moderate model fit, χ^2^(78) = 79.50, *p* = 0.43, CFI = 1.00, TLI = 1.00, RMSEA = 0.09, AIC = 8855.76, BIC = 9000.16. CFI for the present model is above the acceptable threshold of 0.95, as is the case for TFI. In addition, the RMSEA value falls above the threshold of 0.06. Taken together, Benedek et al.’s [19] network model shows moderate model fit. 

**Figure 5:**
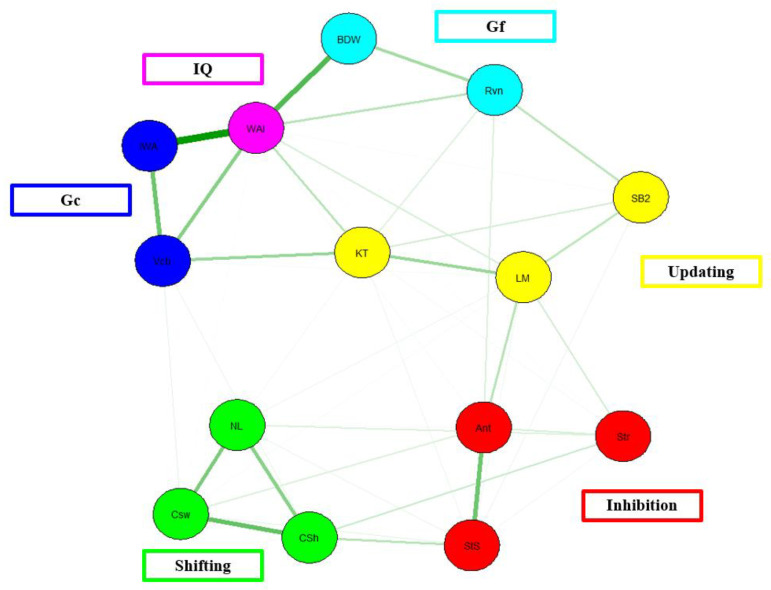
Network Model of Friedman et al. [5] EF data. Note. Blue = Crystallized intelligence. IWA = information WAIS, Vcb = vocabulary, Green = Shifting. NL = number letter, Csw = category switch, Csh = color shape. Red = Inhibition. Ant = anti-saccade, Str = Stroop test, StS = stop-signal. Yellow = Updating. KT = keep track, LM = letter memory, SB2 = spatial 2-back. Teal = Gf. Rvn = Raven’s progressive matrices test, BDW = Block-design WAIS, Pink= Composite IQ scores, WAI= WAIS test battery.

The network model for Friedman et al.’s [5] data (see Figure 5) shows more adequate unity and diversity of EFs. For example, within each EF domain, the tasks representing the same EF construct are more tightly connected (as evidenced by the thicker edges), and there are also numerous connections across EF tasks measuring different EF constructs. However, the strength of the cross-domain EF edges is not strong. 

In terms of model fit, there is poor model fit, χ^2^(43) = 76.79, *p* < 0.001, CFI = 0.97, TLI = 0.94, RMSEA = 0.06, AIC = 8285.97, BIC = 8500.20. CFI for the present model is above the acceptable threshold of 0.95, as is the case for TFI. In addition, the RMSEA value falls below the threshold of 0.06. Taken together, the network model for Friedman et al. [5] data shows an acceptable model fit. 

**Figure 6:**
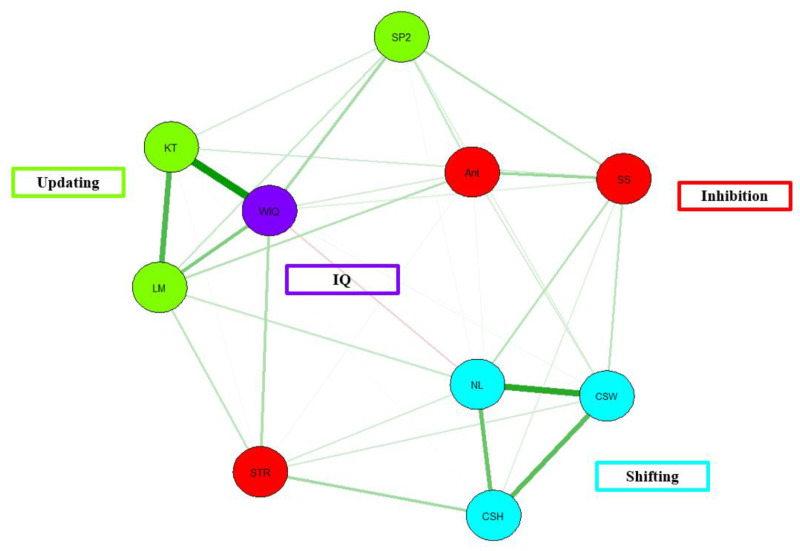
Network Model of Friedman et al. [18] EF and intelligence data. Note. Green = Updating. LM = letter memory, KT = keep track, SP2 = spatial 2-back. Red = inhibition. Ant = anti-saccade, SS = stop-signal, STR = Stroop test. Teal = Shifting. NL = number letter, CSW = color switch, CSH = color shape. Purple = IQ. WIQ = WAIS composite IQ score.

Similar to their earlier study, a network model of Friedman et al.’s [18] data shows similar patterns of edge connections among and between the EF nodes (see Figure 6). However, it is important to note that the inhibition tasks are not strongly converging with one another, showing a lack of construct validity for this EF construct. 

In terms of model fit, there is adequate model fit, χ^2^(10) = 6.20, *p* = 0.80, CFI = 1.00, TLI = 1.00, RMSEA = 0.00, AIC = 25,098.18, BIC = 25,316.48. CFI for the present model is below the acceptable threshold of 0.95, as is the case for TFI. In addition, the RMSEA value falls below the threshold of 0.06. Taken together, Friedman et al. [18] data shows an acceptable model fit.

**Figure 7:**
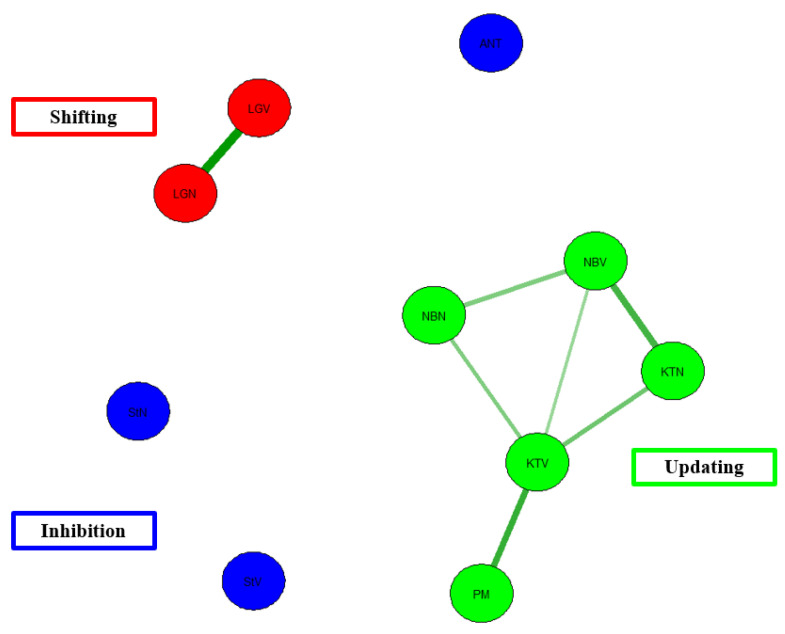
Network Model of Hull et al. [21] EF data. Note. Red = Shifting. LGV = local-global verbal, LGN = local-global non-verbal. Blue = Inhibition. ANT= Antisaccade task, StN = Stroop non-verbal, StV = Stroop verbal. Green = Updating. PM = plus-minus, KTV = keep track verbal, KTN = keep track non-verbal, NBV = N-back verbal, NBN = N-back non-verbal.

Figure 7 shows a network model for Hull et al.’s [21] data. This model shows that there is cohesion among the shifting tasks and cohesion among the updating tasks. These nodes tend to be strongly related to one another. However, this is not the case for inhibition. The inhibition tasks do not show correlations among one another. Again, though there are associations for tasks representing the same EF construct, there are no associations between tasks measuring distinct EFs. For example, there are no associations between inhibition tasks and shifting tasks. Likewise, there are generally no associations (edges) between updating tasks and shifting tasks. This is alarming given the idea that EF should be correlated according to the unity/diversity model of EF. 

In terms of model fit, there is poor model fit, χ^2^(48) = 96.94, *p* < 0.001, CFI = 0.67, TLI = 0.61, RMSEA = 0.12, AIC = 2736.74, BIC = 2781.03. CFI for the present model is below the acceptable threshold of 0.95, as is the case for TFI. In addition, the RMSEA value falls above the threshold of 0.06. Taken together, the network model for Hull et al. [21] data shows poor model fit. 

**Figure 8:**
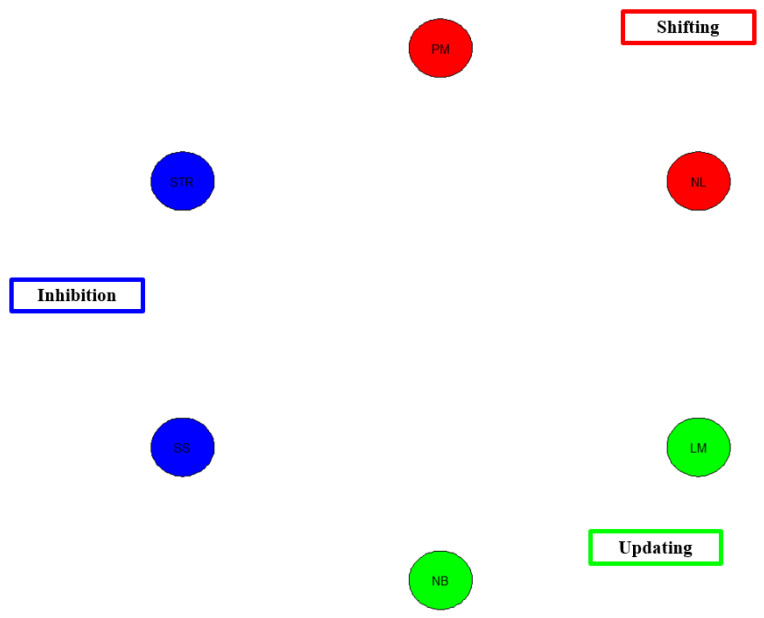
Network model of Del Missier et al. [30] data. Note. Blue = shifting. SS = stop-signal, STR = Stroop test. Red = Shifting. PM = plus-minus, NL = number letter. Green = Updating. NB = N-back, LM = letter memory.

Figure 8 below shows a network model for Del Missier [30]. This model mirrors that of Miyake et al.’s [9] model. Given the low correlations among the EF tasks, there is no network structure for the EF tasks. This finding continues to add to the general pattern of results that the psychometric structure of EFs is not adequate. 

**Figure 9:**
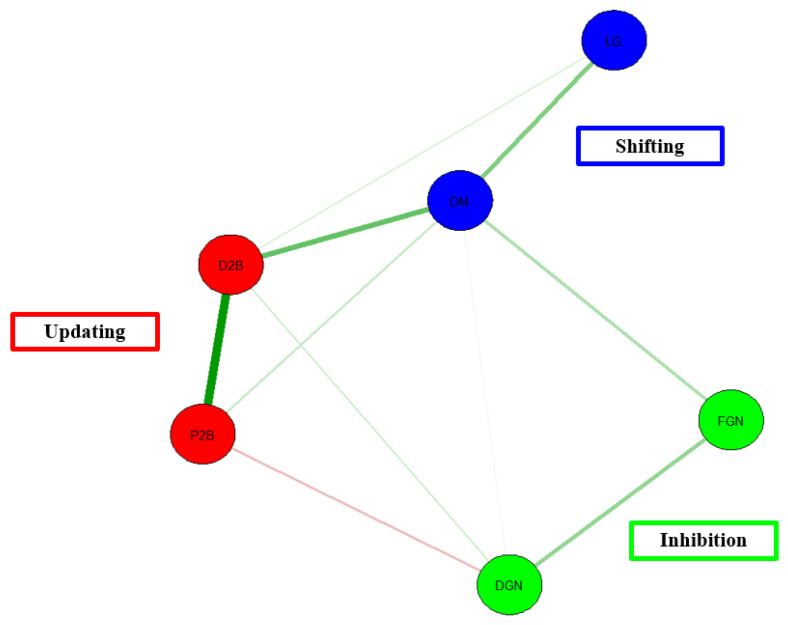
Network Model of Duan et al. [28] data. Note. Red = updating. D2B = Digit 2-back, P2B = Position 2-back. Blue = Shifting. LG = local-global, OM = odd-more. Green = Inhibition. DGN = digit Go/Nogo, FGN = figure Go/Nogo.

Figure 9 below shows a network model of Duan et al.’s [28] data. Overall, the network model shows that there is a convergence of tasks measuring the same EF construct as well as the convergence of tasks across EFs. This network model stands out from the earlier models in this sense. Unlike previous models where there was no unity across EFs, we do find evidence for convergence for these data [28]. For instance, there are some edges shared between the tasks of updating and shifting. Nonetheless, there is still a large absence of edges between the EF tasks. 

In terms of model fit, there is adequate model fit, χ^2^(5) = 4.62, *p* < 0.001, CFI = 1, TLI = 1, RMSEA = 0.00, AIC = 930.71, BIC = 964.49. CFI for the present model is above the acceptable threshold of 0.95, as is the case for TFI. In addition, the RMSEA value falls below the threshold of 0.06. Taken together, the network model for Duan et al. [28] shows an acceptable model fit. 

The central goal of Study 2 was to examine the structure of EF using network analysis. Overall, the psychometric network model results indicate that across the EF data sets, there is more diversity than unity of EFs. Specifically, there is a general lack of convergence between tasks measuring the same construct (as illustrated by the lack of edges between nodes of EF). This is especially true for the inhibition measures across the studies assessed here. These results call into question the psychometric strength of the well-embraced 3-factor measurement model of EFs [9]. A deeper discussion of these findings is presented next. 

## 4. Discussion

Long-standing discussions in the field of cognitive science revolve around the generality versus specificity of EFs (for a review, see Doebel [6]), which have important implications at both the theoretical and applied levels of the field. This dichotomy is encapsulated by opposing arguments where one proposes that executive functions develop as domain-general mechanisms (generality perspective), while the other argument postulates that executive functions operate differently according to specific task demands (specificity perspective). Consistent evidence shows that EF is critical for many outcome variables (e.g., academic achievement, job performance, etc.); therefore, examining the strength of the psychometric model that has long driven applied work in this area (i.e., Miyake et al. [9]) is important. The current project examined whether the popular three-factor model of EF proposed by Miyake and colleagues [9] was psychometrically valid or provided sufficient explanatory power for the structure of EF. Miyake and colleagues [9] posited that EF components in their model (i.e., shifting, updating, and inhibition) were both unified (in that they all explained executive function) and diverse (each component operated individually). Utilizing two studies, the current work aimed to evaluate the psychometric structure of this model using two different types of analyses, as doing so can inform the field and ensure that our understanding of EF captures the complexity of its basic components. 

Results from Study 1 showed the EF components of shifting, updating, and inhibition inadequately explained the variance in the various cognitive tasks. On the one hand, the mean effect sizes for these three components (0.85 for shifting, 0.80 for updating, and 0.55 for inhibition) indicate that across studies, the tasks used to measure shifting and updating were successful at predicting the performance of these EF components, while the tasks for inhibition were less so. However, what is particularly troublesome is the large range of variance explained across the three EF components (72% for shifting, 64% for updating, and 30% for inhibition). While one might argue that the components of the shifting and updating approach explained adequate amounts of variance, the inhibition factor did not, and even in the case of shifting and updating, 28–36% of the variance is still unaccounted for. In addition, the variability across the three components (ranging from 30% to 72%) is problematic.

These findings are troubling for several reasons. First, in considering the outcomes from the inhibition factor only, results demonstrated that the model of inhibition yielded low explanatory power, corroborating other studies with similar results [32]. Specifically, Rey-Mermet et al. [32] tested various models of inhibition across older and younger adults using CFA analyses, finding proportions of explained variance ranging from 2% to 36%, resulting in a conclusion that what is being termed “inhibition” should be reconsidered. A vast amount of research has embraced this construct in predicting real-world outcomes like creativity [19], delayed gratification [33], and academic performance [34]. However, if the factor of inhibition is showing repeated low explanatory power in predicting performance on tasks thought to be tapping into the construct across studies, the currently accepted and prominent model of EFs proposed by Miyake and colleagues [9] should be revised to consider whether the EF factor of inhibition is truly an EF pertaining to the model. If it is not, alternative EF should be considered. Further, empirical investigations are needed to establish what the inhibition tasks are actually assessing. 

Second, while shifting and updating fared better in the findings of Study 1, some questions remain. As with inhibition, shifting and updating are predictive of real-world abilities [5,9,19]. Given the current results, while well over half of the variance (72% for shifting, 64% for updating) in tasks measuring these EF components is explained by the shifting and updating latent factors, there remains a notable amount of variance unexplained. This calls into question whether the psychometric nature of EF for these components is distinct or is a conglomeration of other abilities. For instance, in the case of the updating component, it is likely that the tasks designed to measure updating are also measuring components of working memory capacity, like attention, given that updating is a capability necessary for working memory to function optimally. Because of this, it is probable that, updating tasks used to derive an updating EF factor are also recruiting working memory abilities. 

Miyake and colleagues [9] posited the general idea of unity and diversity in EF. That is, their model showed that although the EF factors and tasks are correlated, they are also separate factors that operate individually. However, the current meta-analysis showed that although there is much diversity (divergent validity) among the factors at the task level, there is minimal unity (convergent validity) amongst the tasks measuring the same EF factor. This raises significant questions about the structure of EF. While these questions have been explored at length at the conceptual level [1], less has been done to examine them at the psychometric level. Arguably, the field’s understanding of the EF construct could be enhanced using network modeling, as this approach allows for recognition of the complexity of the cognitive system.

The current study’s focus on the network modeling approach shows just that. Study 2 utilized network analysis to test Miyake et al.’s [9] psychometric structure of EF. Findings revealed a greatly varied set of results that open the door for even more questions about what EF looks like conceptually. Network models revealed the lack of a network among EF abilities with (a) neither divergence [9,21] nor convergence of EF tasks [9,18], and (b) connections within the EF components but no convergence across EF [19]. Specifically, across the various models, the tasks of inhibition, shifting, and updating showed high divergent validity where the tasks measuring the same constructs were generally related to each other (less so in the case of inhibition); however, and perhaps more alarmingly, there was low convergent validity such that the measures of EF were minimally correlated with one another. In other words, diversity was seen across the components of shifting, updating, and inhibition, but these components together lacked unity.

Taken together, the findings of Study 1 and Study 2 have important implications in a number of areas. First, the unity and diversity model put forth by Miyake and colleagues [9]—which is considered a gold-standard model in the field—is called into question. For quite some time now, cognitive science has considered EF to be made up of three components (i.e., updating, shifting, inhibition) that are both unified and diverse, and this model has been used to demonstrate the importance of EF with regard to quite a number of outcome variables [1]. However, as the current project demonstrates, the latent variable model used consistently demonstrates an inability to explain adequate variance in manifest variables, leaving a significant percentage of variance unexplained. Further, the subjective interpretations and the principle of local independence that characterize latent variable modeling add to the ineffectiveness of this approach in the conceptualization of EF, as cognitive tasks are not process pure and the cognitive abilities tapped within those tasks will often be connected to one another.

Further, related to the first implication, a lack of a coherent understanding of EF has great consequences in the applied sector. Specifically, as cognitive scientists design interventions to help mitigate EF concerns demonstrated by individuals in a variety of settings (e.g., the school setting), it is critical that targeted components are accurate. Intervention work is intricately tied to the theoretical models that guide it, and without a clear conceptualization of the EF construct, it becomes unclear whether mixed findings in intervention research are a result of the interventions themselves or a result of targeting components that are actually not a function of EF. It is critical that the field assess the validity of its constructs to increase the effectiveness of the applied work that follows.

Finally, the results of this project add questions surrounding the conceptualization and measurement of the EF construct. Conceptualization and measurement are intricately intertwined. While some might look at these results and question the validity of the EF construct as a whole, it is alternatively suggested that what is needed in the field of cognitive science is a focus on more accurate and updated psychometric measurement of EF. Rather than relying on measurement that restricts the understanding of construct complexity, more informed analyses—like network modeling—that allow for a more intricate, objective look at how the constructs hang together are warranted. In the field of cognitive science, the probable inability to design “process pure” measures highlights the complexity of EF components. While one measure might be designed to assess inhibition, other EF processes (e.g., attention, working memory) might also be assessed simultaneously. Studies administer different measures, and because of this, the different mixtures of tasks across studies lead to the assessment of other abilities not limited to only the intended EFs. This ultimately leads to the low explanatory power of the three-factor model of EFs at the psychometric level. It is this type of complexity that network modeling can capture, given its numerous statistical and theoretical strengths. Thus, we are not stating here that the unity and diversity model of EF is inherently wrong, as it serves an important purpose in neuropsychology. Rather, from a psychometric standpoint, we are suggesting that the use of network modeling can help bridge statistical models and theoretical models of EF and provide an alternative way of conceptualizing the nature of EF. 

Though the current analysis evaluates the validity and predictive power of the three-factor model of EF, there are limitations to be highlighted. First, a relatively small sample of studies was selected for the meta-analysis. Because of this, the generalizability of the current findings is limited. However, according to the fail-safe N index, it would take 7 studies finding null results to reduce the shifting effect size to 0.50, 8 studies to reduce the inhibition effect size to 0.30, and 6 studies to reduce the updating effect size to 0.50. This indicates that it would take about half of the studies included here showing null results to change the results of these findings, which indicates that the results reported here (though with a relatively small sample of studies) are nonetheless meaningful. Secondly, the effect sizes compiled here showed heterogeneity. Because of this, the current results should be considered with some caution. However, it is important to note that heterogeneity may be driven by the issues discussed above (e.g., different tasks, and lack of process pure tasks). Here it is shown that heterogeneity was in part driven by distinct factors and not by the different age groups. Taken together, these results suggest that the current mean effect sizes across the EF factors are unstable, and thus the conclusions derived here should be taken with caution. However, as noted above, this is likely due to measurement challenges. Last, though network modeling holds important advantages over latent variable modeling, some limitations are worth highlighting. First, network models are successful only if the covariance between variables is large [25]. Second, if the data possesses a high measurement error, then the network structure can be misrepresented and misleading [25]. Finally, given the relative novelty of the network modeling technique in the field of cognitive abilities, there is no standard practice for implementing this technique on cognitive-behavioral data. Despite these shortcomings, network modeling shows important strengths in regard to uncovering the psychometric nature of EF, as shown in these two studies. 

## 5. Conclusions

The current results provide emerging evidence for the idea that the EF of inhibition and updating may not be isomorphic but rather are conglomerates of numerous cognitive abilities. Because of this, the current dominant model of EF yields low explanatory power at the psychometric level and calls into question the veracity of the Miyake et al. [9] model. Future studies implementing the three-factor structure of executive functions proposed by Miyake and colleagues to predict other outcomes should do so with caution. Upcoming studies need to further investigate the model of EF via other modeling techniques, such as network modeling analyses, to begin to uncover the true structure of EF in humans. Doing so can shed important insight into how EF is structured and can be measured in applied settings like education and clinical contexts where measuring EF accurately is of utmost importance. Further, the use of psychometric approaches that can address both the generality and context-specificity of EF (thereby bridging the divide within the field of cognitive science) would be worthwhile.

## Figures and Tables

**Table 1:** Summary of indices obtained in a meta-analysis across three EF factor models.

Model	*K*	Min.	Max.	M_ES_	Z	R^2^	CI	Cochran’s *Q*	I^2^
Shifting	10	0.50	1.33	0.85	16.76 **	0.72	0.75, 0.95	44.38 **	79%
Inhibition	10	0.33	0.79	0.55	14.72 **	0.30	0.47, 0.62	22.83 *	60%
Updating	10	0.50	1.38	0.80	9.66 **	0.64	0.64, 0.97	126.56 **	92%

Note. ** *p* ≤ 0.001 * *p* ≤ 0.01, Total N = 2478 participants across all studies sampled. I^2^ = amount of heterogeneity across studies. N = 10 studies. The Hedges school of meta-analysis was adopted in this study. *K* = total number of studies, Z = Z-test statistic indicating whether the effect size is significantly different from zero by utilizing the weighted mean effect size and standard error, R^2^ = Amount of variance explained in the manifest variables by the latent factors across studies. CI = confidence intervals for each effect size estimate.

**Table 2:** Executive function measures were used across all studies.

EF	Measure
**Inhibition**	Stop-signal, Stroop, Anti-saccade, Anti-cue, Flanker, Go-nogo
**Shifting**	Plus-minus, number-letter, local-global, more-less/odd-even, dimensional change card sort task, color-shape, category switch
**Updating**	Letter memory, n-back, keep-track, tone-monitoring, n-back non-verbal, corsi blocks design task

Note: Correlational matrices data were used from the paradigms above.

## Data Availability

The data presented in this study are available on request from the corresponding author.
